# *Erannis jacobsoni* disturbance detection based on unmanned aerial vehicle red edge spectral features

**DOI:** 10.3389/fpls.2025.1619695

**Published:** 2025-09-01

**Authors:** Liga Bai, Xiaojun Huang, Ganbat Dashzebeg, Mungunkhuyag Ariunaa, Shan Yin, Yuhai Bao, Gang Bao, Siqin Tong, Altanchimeg Dorjsuren, Enkhnasan Davaadorj

**Affiliations:** ^1^ College of Geographical Science, Inner Mongolia Normal University, Hohhot, China; ^2^ Inner Mongolia Key Laboratory of Mongolian Plateau Geographical Research, Inner Mongolia Normal University, Hohhot, China; ^3^ Inner Mongolia Key Laboratory of Disaster and Ecological Security on the Mongolia Plateau, Inner Mongolia Normal University, Hohhot, China; ^4^ Institute of Geography and Geoecology, Mongolian Academy of Sciences, Ulaanbaatar, Mongolia; ^5^ Institute of Biology, Mongolian Academy of Sciences, Ulaanbaatar, Mongolia

**Keywords:** *Erannis jacobsoni*, boreal coniferous forest, UAV, red edge features, machine learning, spatial distribution

## Abstract

Boreal coniferous forests play important roles in global ecological and economic processes. Mongolia, rich in forest resources and part of the boreal ecosystem, faces significant deforestation due to *Erannis jacobsoni* Djak (Lepidoptera: Geometridae), a rapidly spreading needle pest in coniferous forests. This study aims to provide with rapid and precise pest occurrence data, enabling timely and effective control measures to preserve and enhance the agroforestry ecological environment. In vegetation disturbance detection, UAV remote sensing exhibits operational performance with unique spatiotemporal advantages (notably cm-resolution data acquisition and flexible revisit cycles) unattainable through traditional ground surveys or satellite platforms. Therefore, we used unmanned aerial vehicle (UAV) imagery from representative areas affected by *E. jacobsoni*, calculated conventional and red edge spectral indices, extracted features sensitive to pest infestation levels, detected disturbances using machine-learning algorithms, and analyzed the pest’s spatial distribution. The sequential forward selection (SFS) and successive projection algorithms (SPA) can effectively extract features sensitive to the response to pest disturbance, in which the red edge (RE) features have a greater effect than the conventional (CONV) features in detecting the pest. The detection models developed using machine learning all achieved accuracy rates above 82%, with the Back Propagation Neural Network (BPNN) performing the best. Spatial distribution analysis based on the model revealed that *E. jacobsoni* primarily exhibited a pattern of outward diffusion from the center of aggregation during the outbreak period.

## 1 Introduction

Coniferous taxa are distributed in most vegetation biomes worldwide (Rautiainen et al., 2018). Of these, boreal coniferous forests play an important part in terrestrial ecosystems (Zheng et al., 2023), serving as biodiversity refuges and major CO_2_ filters (Diez et al., 2021), with large pools of atmospheric CO_2_ stored in their organisms and soils (Ciais et al., 2013; Duarte et al., 2022; Yang et al., 2023). They also hold economic value through softwood production and petroleum substitutes, making them vital to global ecological and economic processes (United Nations Economic Commission for Europe/Food and Agriculture Organization of the United Nations (UNECE/FAO), 2016; FAO and UNEP, 2020). Intensifying climate change has disrupted natural disturbance cycles, including wildfires and pest outbreaks, significantly affecting coniferous forests (McCullough et al., 1998; Ramsfield, 2016; Seidl et al., 2017). Among these, the increasing frequency and scale of pest outbreaks in forests worldwide have garnered significant attention (Seidl et al., 2016; Jactel et al., 2019; Przepióra et al., 2020). According to reports, between 2002 and 2010, an average of 14.5 million m^3^ of timber in Europe was affected annually by bark beetle infestations (Sommerfeld et al., 2021); in temperate forests of North America, the area impacted by pests and diseases each year was nearly 50 times that of wildfires (Dale et al., 2001); from 2003 to 2012, approximately 85 million hectares of global forest were disturbed by pests (van Lierop et al., 2015); and most global climate change scenarios favor an increase in the outbreak rates of pests in temperate forests in the future (Logan et al., 2003). This indicates that the health of coniferous trees will be compromised (Gromtsev, 2002), and the cyclical processes of coniferous forest ecosystems will face more significant disruptions.

Mongolia is rich in forest resources, amounting to 18.6 million hectares, of which 14.2 million are coniferous forests. These are an important part of the Mongolian and boreal forest ecosystems (FAO, 2020; Tungalag et al., 2020). Since 1980, approximately 11.52 million hectares of forests in Mongolia have been infested by pests, resulting in a 0.39% decline in forest cover, 30% forest degradation, and 17% of forests classified as severely damaged and at risk of extinction (Ma et al., 2022). Additionally, forestry department statistics show that between 2016 and 2020, pest infestations affected 1.79 million hectares. Among these, *Erannis jacobsoni* Djak (Lepidoptera, Geometridae) is one of Mongolia’s most widespread and characteristic conifer pests. It was first identified in Russia’s South Siberian region by Soviet scholar Djakonov (Djakonov, 1926). Subsequently, in 1929, another former Soviet scholar, Viedalep, observed that the pest had spread to Mongolia’s Zavkhan Province (Viedalep, 1975). Currently, its outbreaks are primarily concentrated in the northern forests of Mongolia. The *E. jacobsoni* poses a severe threat to forest trees. Its larvae feed on larch needles between May and June, causing a gradual reddening of the forest’s appearance or tree death, resulting in a generalized decline in coniferous forests (Bai et al., 2021). This pest is highly adaptable to the environment and rapidly becomes a dominant species once it invades (Huang and Bao, 2017). Since 2010, *E. jacobsoni* infestations in Mongolia’s larch forests have expanded from tens of thousands to hundreds of thousands of hectares, significantly impacting coniferous forest ecosystems (Ma et al., 2022).Traditional pest control in Mongolia relies on manual field surveys and monitoring records, which are limited by weather, terrain, and time delays. These limitations hinder large-scale monitoring of *E. jacobsoni*. It also involves a time lag and lacks timeliness in specific surveys, resulting in delayed transmission of outbreak information. Therefore, there is an urgent need for technologies capable of meeting both spatial and temporal requirements to perform large-scale, accurate monitoring of *E. jacobsoni* pests. This will provide necessary information on the pest situation to the relevant forestry departments, and to achieve effective maintenance of the balance of the ecosystem of coniferous forests.

When a tree is infested by a pest, its appearance and physical and chemical indicators, such as chlorophyll, nitrogen, and moisture content, are abnormal, causing the spectral reflectance to rise and fall (Imran et al., 2020; Jiang et al., 2021; Lv et al., 2024). This makes it a key tool for pest and plant health monitoring, offering potential for real-time assessment of pest-induced damage. Over the past three decades, remote sensing technologies have rapidly advanced and become increasingly prominent in plant health monitoring. Among these, unmanned aerial vehicle (UAV) remote sensing is widely favored by researchers (Brovkina et al., 2018; Wang et al., 2022). UAV has some advantages over traditional monitoring methods, including: (1) Lower labor intensity, (2) Reduced time costs, (3) Flexible sampling intervals, (3) monitoring of diverse and even hardly accessible habitats (Garcia, 2020). As a low-altitude remote sensing platform, UAVs can acquire higher-resolution imagery compared to satellite remote sensing and enable short-notice revisits to target areas as needed. This effectively solves the problem of frequent data gaps in satellite imaging caused by adverse weather conditions such as clouds, rain, and fog (Lu et al., 2022), making it especially valuable for small and medium scale research (Banu et al., 2016; Senthilnath et al., 2017). Equipped with highresolution sensors, the device offers strong potential for canopy-scale pest detection (Yang et al., 2017; Yu et al., 2021). Among these, airborne multispectral sensors have been widely adopted in the UAV industry due to their advantages in cost, operation, size, and weight (Eugenio et al., 2021). Integrating it with traditional surveys would help minimize the cost of pest monitoring (Duarte et al., 2022). Huang et al. (2018) combined UAV-based multispectral sensor-collected images and ground survey data to detect the level of spider mite infestation. Marston et al. (2019)detected stress on farmland caused by Soybean Aphid infestation through UAV multispectral imagery. Zhang J. et al. (2018) targeted the Yunnan pine forest area invaded by the bark beetle *Tomicus yunnanensis* (Coleoptera, Curculionidae, Scolytinae) and achieved high-precision pest area identification based on UAV multispectral images. Most studies have applied UAV multispectral applications to the detection of agricultural diseases, weeds, and forest borer pests. Among these, positioned between the red band, associated with strong chlorophyll absorption, and the near-infrared band, linked to light scattering in the leaf mesophyll, the red edge band is sensitive to vegetation growth conditions, and physiological and biochemical parameters. Therefore, it is the most informative and important band reflecting the plant’s growth and cover (Huang et al., 2003; Delegido et al., 2013; Jin et al., 2014). It has been used in some vegetation health monitoring studies and demonstrated superior performance compared to conventional features (Bhattarai et al., 2020). Therefore, employing red edge related-features for detecting *E. jacobsoni* damage levels is likely to yield optimal results and enhance control efforts.

In plant pest research, appropriate features play a critical role in subsequent detection and classification. Therefore, it is crucial to select remote sensing indicators that are highly responsive to pest damage and integrate them with effective classification models to enhance detection performance (Duarte et al., 2022; Xu et al., 2022a; Shi et al., 2024). Among feature screening algorithms, analysis of variance (ANOVA), which evaluates the impact of different factors on data variability (Kim and Park, 2018) and importance metrics, which quantify the contribution of features to model performance (Duarte et al., 2020) have gained widespread attention (Duarte et al., 2022). The successive projection algorithm (SPA) is a method that minimizes collinearity between variables through iterative projections.It has been applied and achieved positive results in previous studies on the pest infestation of *E. jacobsoni.* For instance, Xi et al. (2022) extracted hyperspectral features sensitive to needle chlorophyll and water content from *E. jacobsoni*-damaged trees, Ma et al. (2022) identified multispectral and texture features responsive to *E. jacobsoni* outbreaks, while Bai et al. (2024)successfully extracted RGB features sensitive to *E. jacobsoni* occurrence. In terms of classification models, many algorithms have successfully detected symptoms in disturbed plants (Ecke et al., 2022). The Random Forest (RF), which integrates multiple decision trees, performs exceptionally well in geographic objects-based small-sample image analysis, and Duarte et al. (2020) successfully used this algorithm to detect damage caused by Eucalyptus Longhorned Borers. The Back Propagation Neural Network (BPNN), based on the chain rule and gradient descent principles, is highly popular for predicting complex nonlinear systems, and Ma et al. (2019) applied this method to identify powdery mildew and aphids in winter wheat, achieving an accuracy of 82.6%. The Convolutional Neural Network (CNN), due to its ability to automatically learn features, exhibits excellent performance in processing one-dimensional spectral data, and He et al. (2025) employed this algorithm to detect the severity of damage caused by Pantana phyllostachysae Chao and obtained satisfactory results.

Different pests exhibit significant variations in spatial distribution patterns and damage symptoms (Kozár et al., 1994; Stone and Mohammed, 2017). Although the application of UAV-based red edge indices in vegetation detection is advancing, research on their use for geometrid pests remains unclear, particularly in assessing responses to damage severity levels and characterizing spatial distribution patterns. This gap constitutes a critical research priority. Based on this, we posit the underlying hypothesis: red edge-related (RE) features can effectively quantify pest infestation severity and characterize spatial distribution patterns. The objectives of this study were to (I) examine whether the RE feature has an advantage over the conventional (CONV) feature in monitoring the disturbance of *E. jacobsoni*, (II) compare the effectiveness of several feature selection methods and machine-learning models in the detection of pests, and (III) obtain the spatial distribution pattern of pest disturbance. Using UAV multispectral imagery from Binder, Khentii Province (a representative E. jacobsoni outbreak zone), we screened sensitive features through ANOVA, SPA, and the importance-based sequential forward selection (SFS) method, and assessed damage levels using machine learning algorithms. The findings provide empirical insights and scientific references for large-scale forest pest management.

## 2 Materials and methods

### 2.1 Experimental site

**Figure 1:**
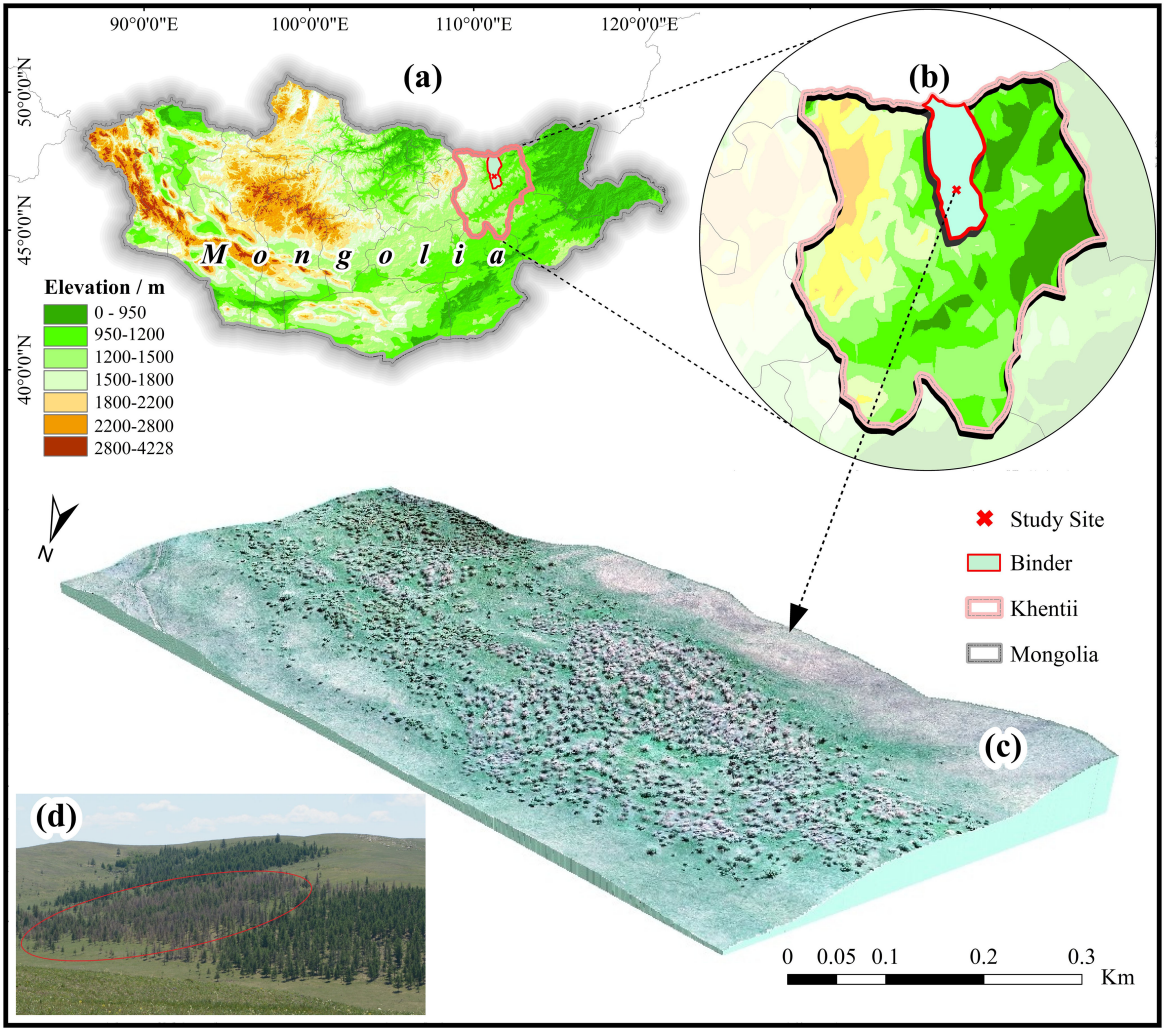
Study area for *Erannis jacobsoni* disturbance detection in 2020. **(a)** Specific geographical locations of study area in Mongolia; **(b)** Study site location (X) in Binder Soum (red border), Khentii Province (pink border); **(c)** 3D visualization of the experimental area generated by integrating UAV aerial photography with Digital Elevation Model (DEM); **(d)** Field photographs of the experimental area, with the damaged trees marked in red circles.

The study area was located in a typical area of *E. jacobsoni* infestation in Binder, Kentii Province, Mongolia, as a forest area 600 m long and 300 m wide, with central coordinates at 110°46’16.616”E, 48°26’25.667”N, and has an average elevation of 1,182m (**Figure 1**). The vegetation is predominantly deciduous coniferous forest, primarily consisting of Siberian larch (*Larix sibirica*) trees ranging in height from approximately 6 to 20 meters, with a very sparse admixture of birch (*Betula platyphylla*) and minimal understory shrub cover. This relatively simple forest structure is less resistant to pest invasion and thus suitable for the occurrence of *E. jacobsoni* (Rigot et al., 2014). Through on-site investigation in early June 2020, the area was severely infected with E. Jacoboni and became a dominant species, with no observable signs of other pests, wildfire or drought. Orthophoto imagery was subsequently acquired via UAVs on June 20, serving as the primary dataset for detection research.

### 2.2 Data preprocessing

#### 2.2.1 Field survey data

Field data collection was conducted within five days before and after the UAV flight. We evaluated damage levels in 840 sample trees using the canopy leaf loss rate (LLR), resulting in a distribution of 210 trees each in the healthy, mild, moderate, and severe categories. The spatial distribution and images of trees at each level are shown in **Supplementary Figures S1A** and **B**. The methodology is as follows: Multiple staff members observed several branches deemed representative of each sample tree’s condition. After consolidating their observations, we selected nine sample branches for counting healthy and damaged needles. The LLR was then calculated for each tree using the following formula (Huang et al., 2019):


$$LLR_{i}=\frac{L_{di}}{L_{hi}+L_{di}}\times 100\%$$

Where LLRi denotes the relative leaf loss rate of the *i*th sample tree, which ranges from 1 to 100%, and $L_{hi}$ and $L_{di}$ are the numbers of healthy and damaged needles in the *i*th sample tree, respectively. On this basis, the level of damage to each sample tree was evaluated, with the association between the LLR and the level of damage (Ma et al., 2022; Bai et al., 2024).

#### 2.2.2 UAV remote sensing data

UAV aerial photography, conducted with a field survey, utilized a DJI Phantom 4 multispectral quadcopter drone (**Supplementary Figure S1C**) with a 5-band multispectral sensor (blue, green, red, red edge, near-infrared) offering 200-pixel and centimeter-level resolutions. Data was collected under clear, cloudless, windless conditions from 10:00 to 14:00 in Beijing, at a 100 m altitude and 8 m/s speed. The camera, calibrated with a whiteboard pre-flight, was directed vertically downward during capture. Images were pre-processed in DJI Terra with reflectance and geometric corrections, and ArcGIS 10.3 overlaid them with ground survey data to map ROIs of sample tree crowns, minimizing shadow and vegetation effects, for extracting tree features.

#### 2.2.3 Feature calculation

Spectral vegetation indices, which are combinations of two or more reflectance wavelengths, can enhance the differences in reflectance between stands at various levels of damage and are less affected by light and the background. Among these, the RE indices are advantageous for monitoring vegetation stress (Runesson, 1991; Abdullah et al., 2019; Liao et al., 2022; Ma et al., 2022). In this study, 62 vegetation indices, commonly used in vegetation monitoring research, were selected as features to detect the damage level of *E. jacobsoni* (**Supplementary Table S1**), including 31 RE index features (Tian et al., 2020; Yang et al., 2020; Niu et al., 2021; Sa et al., 2024). The ENVI Bandmath module was used to calculate the vegetation index for each sample ROI stepwise, with the average value of each ROI serving as the eigenvalue for the corresponding sample tree.

### 2.3 Data analysis

**Figure 2:**
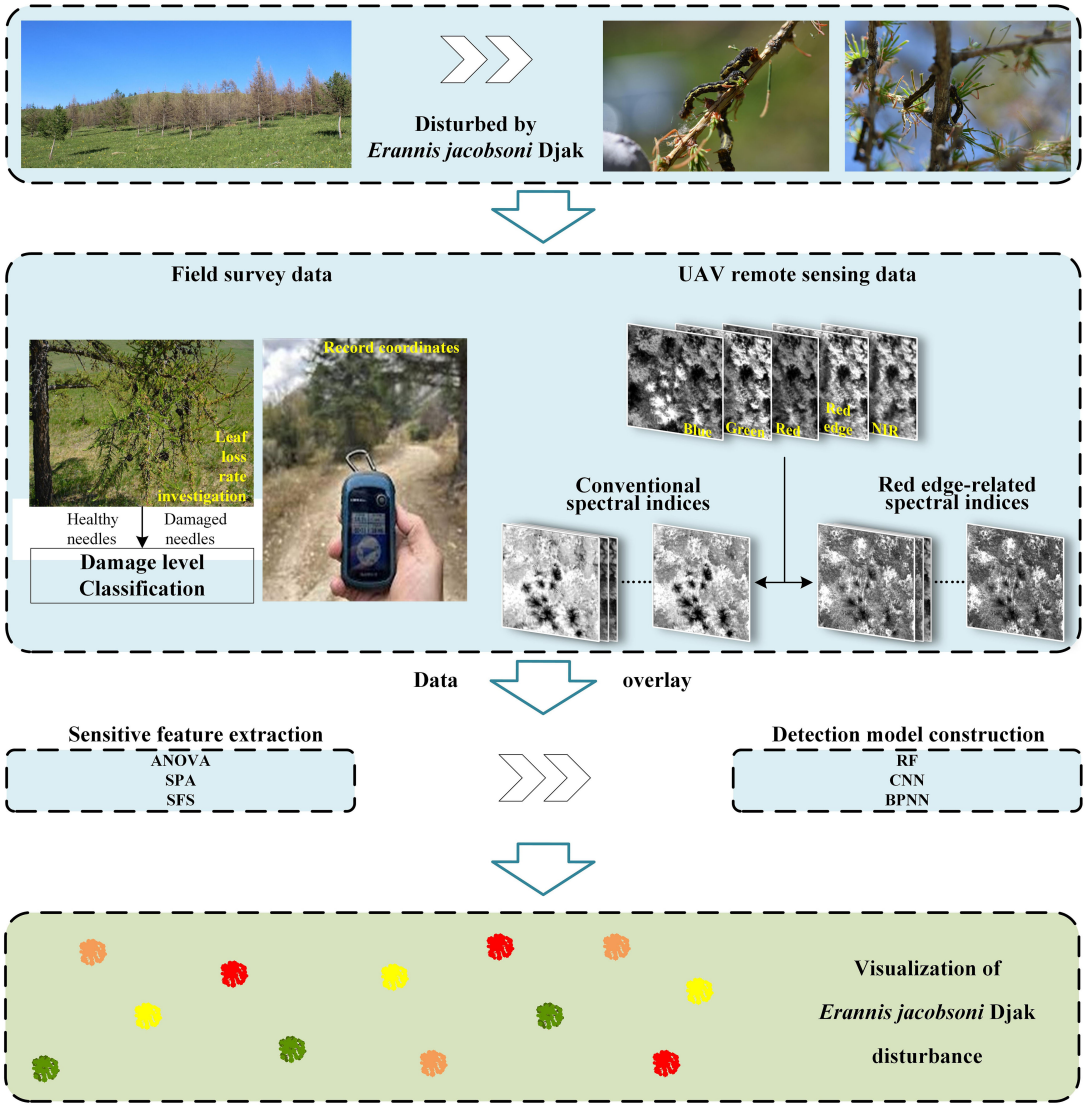
Method pipeline of *Erannis jacobsoni* disturbance detection.

Here, we analyzed the response of spectral indices to *E. jacobsoni* damage levels by integrating field-assessed damage ratings of sample trees with spectral feature data obtained from the experimental area. We extracted the sensitive feature set using the ANOVA, SPA, and SFS algorithms, and conducted a study on the detection of the *E. jacobsoni* disturbance using three algorithms, namely, RF, CNN, and BPNN. The overall methodology pipeline is shown in **Figure 2**.

#### 2.3.1 Sensitive feature extraction

In classification tasks, all types of algorithms benefit from feature space downscaling to reduce the risk of overfitting and improve model accuracy (Guyon et al., 2002). Therefore, three dimensionality reduction algorithms were used in this study to filter features sensitive to pest damage variation.

1. Analysis of variance.

ANOVA is a technique used to analyze the extent to which the mean values of variables are affected by different types and combinations of factors (Bewick et al., 2004). By calculating the F-value between pest damage levels and the original features through ANOVA, the variation between classes can be determined, identifying characteristics that significantly respond to the target variable.

2. Successive projection algorithm.

The SPA is a forward variable selection algorithm that uses projection operations to minimize covariance among modeling variables by selecting those with the largest projection in the orthogonal subspace of previously chosen variables (Zhang N. et al., 2018). This eliminates redundant information in vegetation indices, allowing the extraction of a few independent features to improve model accuracy (Huang et al., 2019; Liu and Guo, 2015).

3. Sequence forward selection.

SFS is a feature selection algorithm that enhances model accuracy and prevents overfitting by selecting the optimal subset of features (Işık, 2020). It ranks features based on their importance for detecting pest damage levels using mean decrease in accuracy, then iteratively adds the most important features to the model, evaluating accuracy each time, until all features are included. The combination with the highest accuracy and fewest features is chosen as the input for the pest detection model (Zhu et al., 2009; Du et al., 2023).

#### 2.3.2 Model building

In this study, the sensitive feature subset, comprising sensitive indicators from both CONV and RE feature sets, is regarded as the explanatory variable. The damage level of *E. jacobsoni* is detected in MATLAB based on three models, that is, the traditional machine-learning model RF and the deep learning models CNN and BPNN.

1. Random forest.

RF is an ensemble learning algorithm that constructs multiple independent decision trees, each trained on a bootstrapped sample and a random subset of features from the original dataset. The final classification is determined by majority voting across all trees, enhancing accuracy and generalization compared to individual decision trees (Torres and Qiu, 2014; Pádua et al., 2020).

2. Convolutional neural network.

A CNN is a feed-forward neural network that uses convolutional layers to extract features. It typically consists of input, convolutional, pooling, fully connected, and output layers (Yan et al., 2021). The convolutional layers extract local features using various kernels, pooling layers filter features and reduce parameters (commonly via max or average pooling), fully connected layers integrate features, and the output layer classifies the data (Kang et al., 2024). CNNs map high-dimensional nonlinear data to a low-dimensional space, reducing parameters and mitigating overfitting (Ma et al., 2022). In this study, a 1D-CNN was chosen for pest damage detection using spectral data (Hu et al., 2015).

3. Backpropagation neural network.

A BPNN is a multilayer mapping network with an input layer, one or more hidden layers, and an output layer, using a structure learning algorithm to minimize backward error during forward information transmission (Liu et al., 2013). It features a simple structure, ease of construction, fast computation speed, and effectiveness in tackling nonlinear problems (Zhu et al., 2019). Trained through supervised learning with backpropagation, it employs activation functions—typically the sigmoid function (ranging from 0 to 1)—to scale neuron outputs before passing them to the next layer (Geethanjali et al., 2008; Zin et al., 2015). This process aims to minimize cumulative error in the training set and maximize recognition performance (Chen et al., 2022).

#### 2.3.3 Model evaluation

In this study, the dataset was randomly split into training and test sets at a 3:1 ratio. The detection model was constructed using the training data and optimized via 5-fold cross-validation. Model performance was evaluated using the test data with metrics including overall accuracy (OA) (ratio of correctly classified samples to total samples, ranging from 0 to 1), kappa coefficient (proportion of error reduction over random classification, typically 0 to 1), recall (ratio of retrieved to relevant samples), F1 score (harmonic mean of precision and recall, balancing specificity and sensitivity) (Zhang X. et al., 2019; Xu et al., 2021), and the confusion matrix (providing user precision (UA) and producer precision (PA) by comparing predicted and actual damage levels). These metrics, derived from true positives (TP), false positives (FP), true negatives (TN), and false negatives (FN), assess the model’s classification accuracy across damage levels. The detailed formulas are as follows:


$$OA=\frac{TP+TN}{TP+TN+FP+FN}\times 100\%$$

$$Kappa=\frac{OA-\frac{\sum_{i=1}^{k}N_{P}\times N_{t}}{S^{2}}}{1-\frac{\sum_{i=1}^{k}N_{P}\times N_{t}}{S^{2}}}$$

$$Recall=\frac{TP}{TP+FN}$$

$$F1=\frac{2TP}{2TP+FP+FN}$$

where *k* is the number of categories, $N_{p}$ is the number of predictions, $N_{t}$ is the number of measurements, and *S* is the sample size.

## 3 Results

### 3.1 Feature sensitivity analysis and extraction

#### 3.1.1 Sensitivity analysis

**Figure 3:**
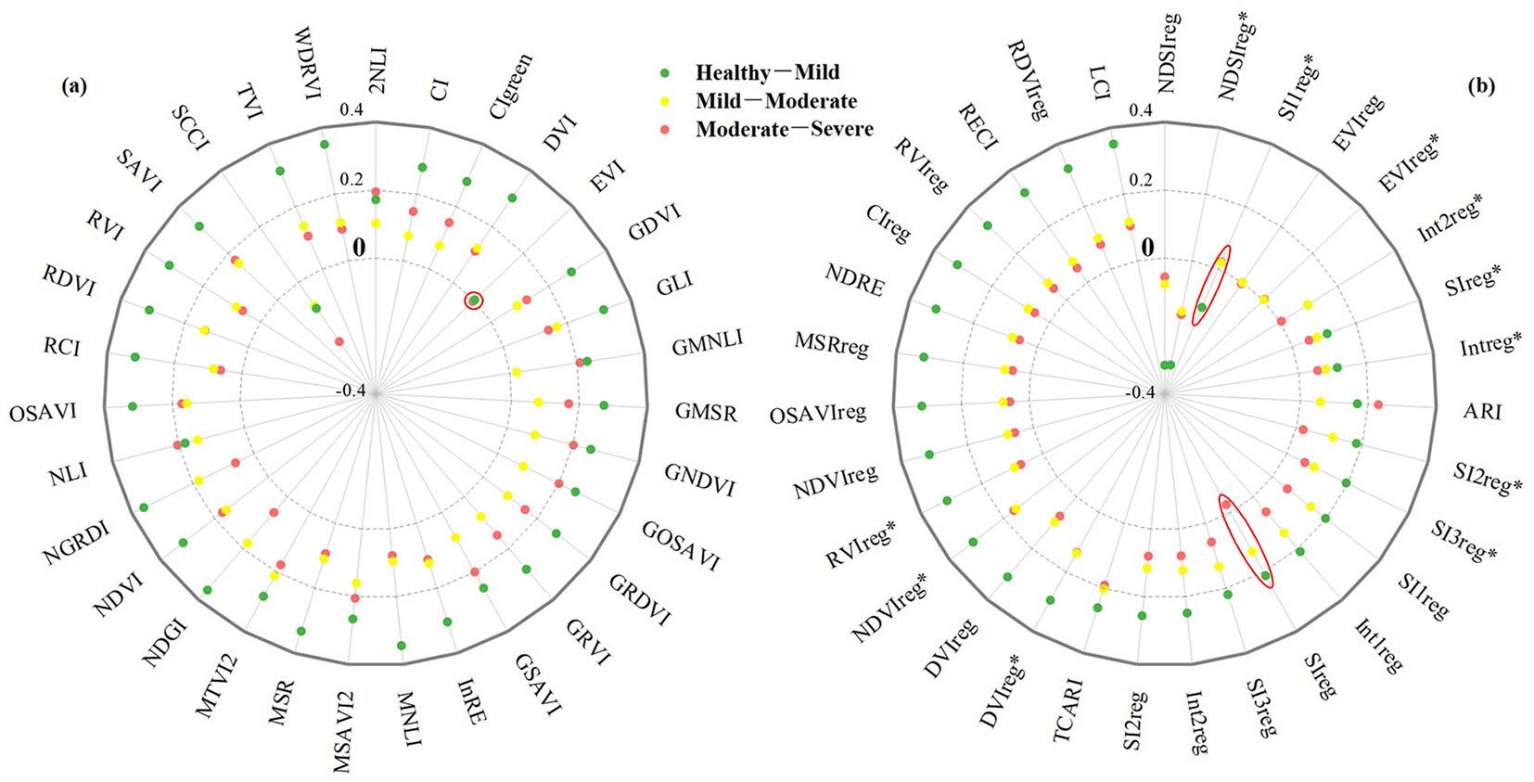
Mean variations of features across *Erannis jacobsoni* damage levels, with red circles highlighting features exhibiting low responsiveness to damage progression. **(a)** Conventional features; **(b)** red edge features.

The distribution of the mean differences in eigenvalues among the damage classes was plotted to determine whether the selected original vegetation indices were responsive to changes in the level of pest damage (**Figure 3**). As shown in the Figure, except for EVI, SIreg, and SI1reg*, which showed a weaker response to changes in damage level (EVI had a positive difference of 0.0019 and 0.0013 in healthy–mild and mild–moderate, and a negative value of −0.0056 in moderate–severe. SIreg showed positive differences of 0.2119 and 0.1308 between healthy–mild and mild–moderate levels, respectively, and a negative difference of −0.0272 between moderate–severe. SI1reg* was negative −0.1235 in healthy–light and positive 0.0204 and 0.0227 in mild–moderate and moderate–severe). All other index features exhibited consistent trends, indicating the selected vegetation indices are promising for pest detection. Among them, SCCI, EVIreg, NDSIreg, and NDSIreg* increased steadily with rising damage levels from healthy to mild, moderate, and severe. Meanwhile, the remaining indices showed a gradual decline.

#### 3.1.2 Sensitive features extraction

To obtain features that were sensitive to changes in the level of pest damage, a subset of sensitive RE features and a subset of sensitive CONV features were filtered from the original set using ANOVA, SPA, and SFS, respectively, and were used as the input variables of the pest detection model.

1. ANOVA-based feature selection results.

**Figure 4:**
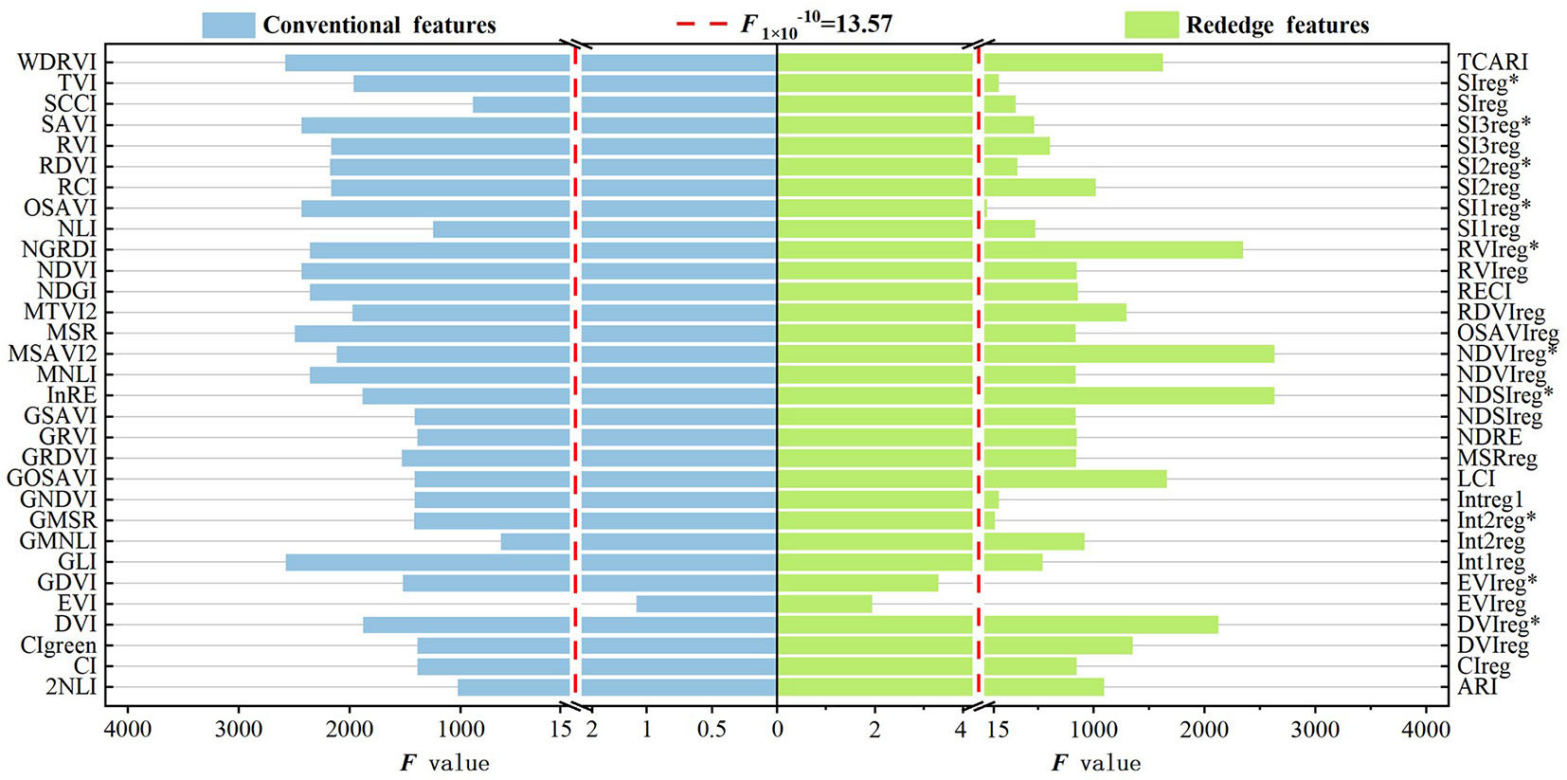
Screening plot of conventional and red edge features for modeling the detection of *Erannis jacobsoni* damage levels using ANOVA. Features with F-values exceeding the sensitivity threshold (red dashed line) are selected as sensitive features.

The ANOVA-based variance distributions of the CONV and RE features are shown in **Figure 4**. The feature statistic F-value is greater than a given threshold value of p < 1 × 10^-10^, indicating that features with F greater than 13.57 are highly significantly sensitive to the level of pest damage. Therefore, features with F-values below the threshold of 13.57 were excluded, and the remaining features were retained as the sensitive feature subset. Among the CONV features, only EVI was excluded due to its low F-value of 0.1898. In contrast, all other CONV features had F-values exceeding 500 and were selected as sensitive features. Among the RE features, the F-values of EVIreg and EVIreg* were 1.4689 and 3.6037, respectively. Therefore, they were excluded, while the F-values of the rest of the features were all higher than 40 and were selected as sensitive RE features.

2. SPA-based feature selection results.

**Figure 5:**
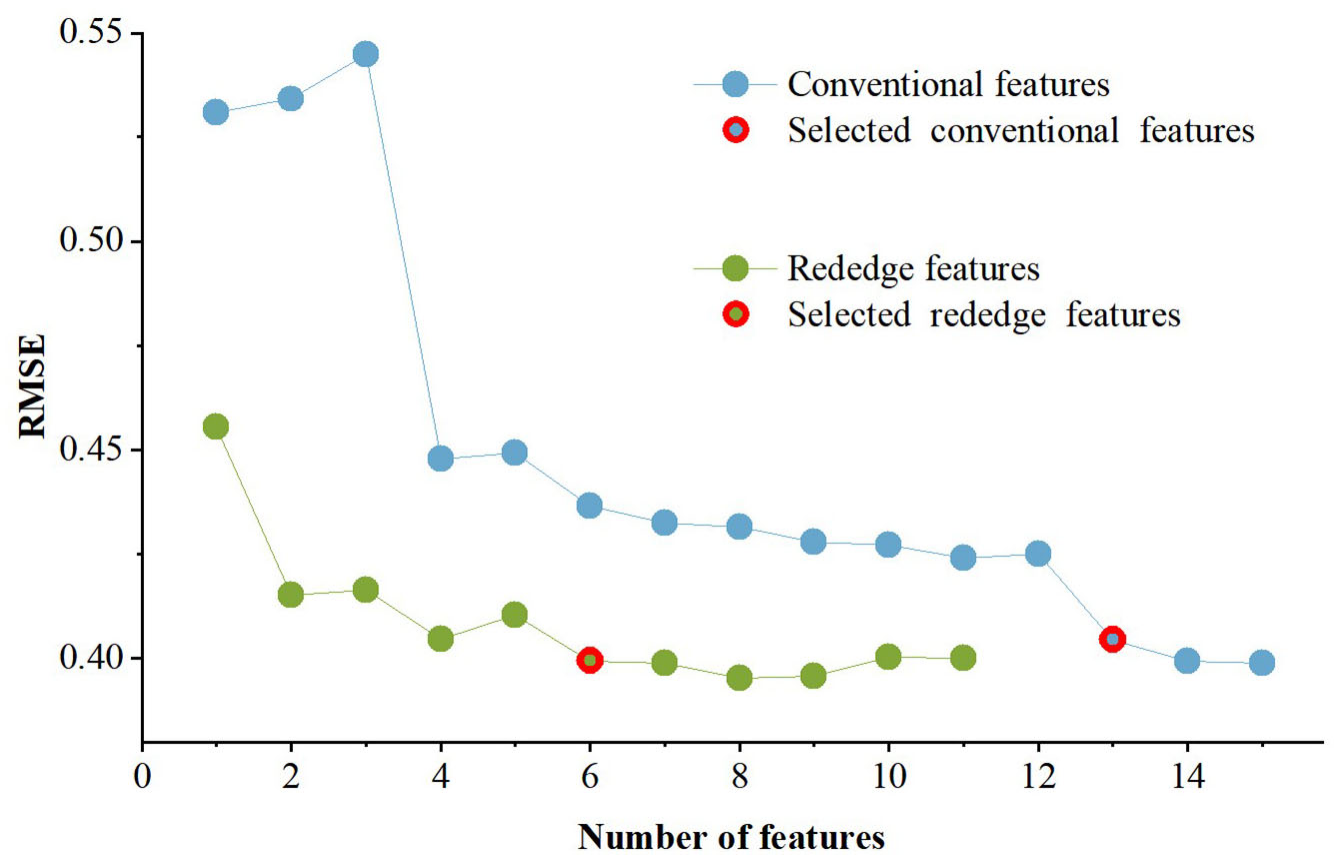
Feature quantity and corresponding RMSE of SPA-screened conventional and red edge features for *Erannis jacobsoni* damge levels detection modeling.

The number of features and the corresponding RMSE based on the SPA are shown in **Figure 5**. In both the CONV and RE feature sets, the RMSE values decreased as the number of features increased, reaching near-minimum values of 0.4044 and 0.3995 at 13 and 6 features, respectively. Beyond these points, further increases in features yielded diminishing RMSE reductions. Therefore, the algorithm uses the principle of minimum redundant information and minimum RMSE among features (Zhang et al., 2022), and selects 13 features (2NLI, CI, GDVI, GLI, GMNLI, GSAVI, InRE, MNLI, MTVI2, NLI, RVI, TVI, and WDRVI) and six features (ARI, DVIreg, Int2reg*, NDSIreg*, SIreg, and TCARI) as sensitive feature subsets in the CONV and RE features, respectively.

3. SFS-based feature selection results.

**Figure 6:**
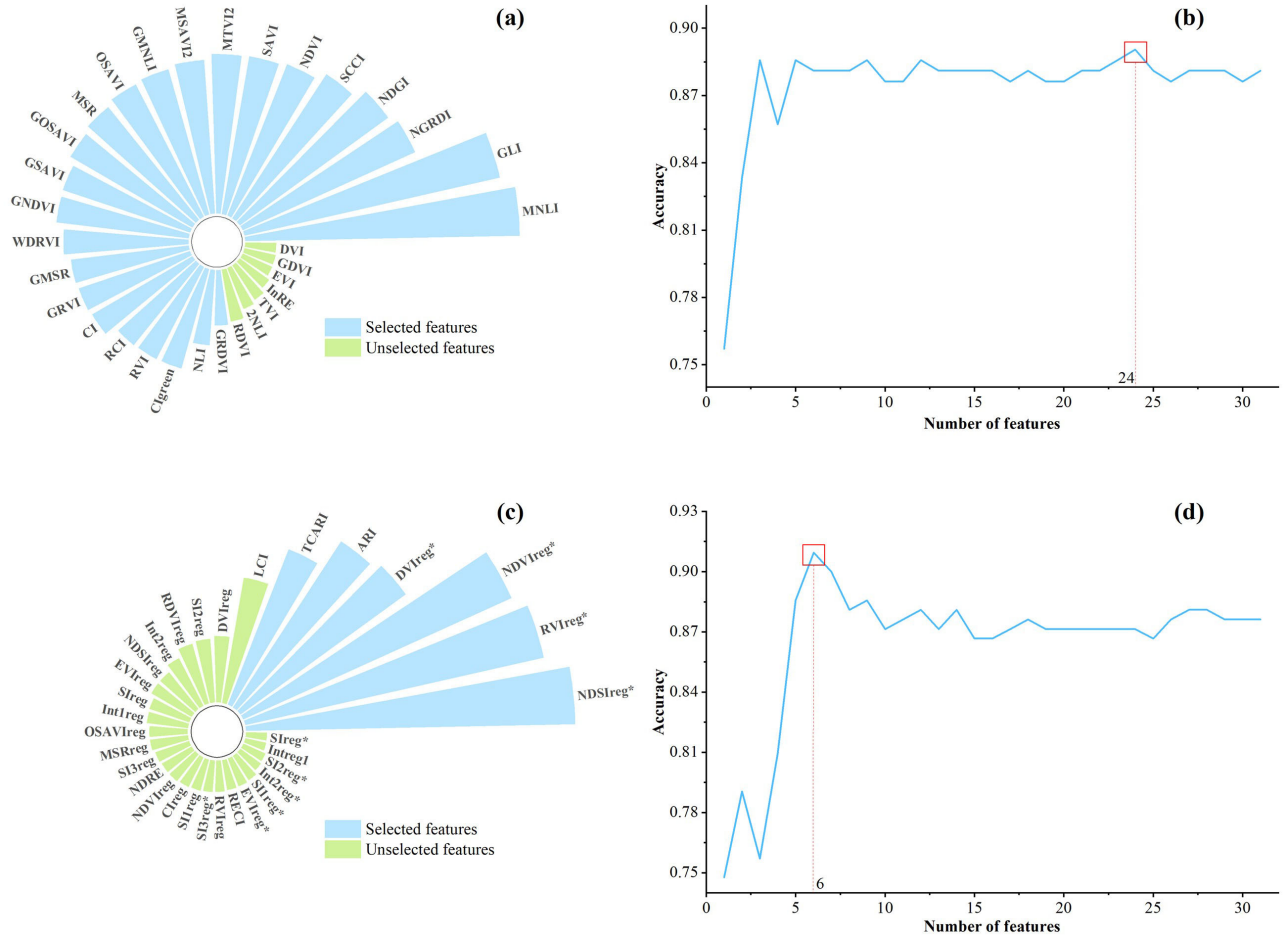
SFS-screened conventional and red edge features for *Erannis jacobsoni* damge levels detection Modeling. **(a)** Importance of conventional features. **(b)** Quantity and accuracy of selected conventional features. **(c)** Importance of red edge features. **(d)** Quantity and accuracy of selected red edge features.

After ranking the importance of the original feature set using RF (**Figures 6a, c**), the number of features based on SFS and the corresponding accuracy varied, as shown in **Figures 6b, d**. For the CONV features, the accuracy increased rapidly with an increase in the number of features until the accuracy tended to be stable when it reached five. The classification accuracy reached its peak at 0.8905 when the number of features increased to 24. Accordingly, 24 features were selected as the sensitive subset of CONV features. These included the majority of features, excluding RDVI, 2NLI, TVI, lnRE, EVI, GDVI, and DVI, all of which had importance values below 0.017. In contrast, for the RE feature set, the accuracy improved markedly with the addition of features, peaking at 0.9095 when the number of features reached six, after which it leveled off. Six features were selected as a subset of the sensitive RE features, including NDSIreg*, RVIreg*, NDVIreg*, DVIreg*, ARI, and TCARI, with the importance of all being higher than 0.06.

### 3.2 Model accuracy

Based on the subset of sensitive RE features screened by the three data dimensionality reduction methods, a model for detecting the damage level of *E. jacobsoni* was constructed with the help of RF, CNN, and BP algorithms and compared with the CONV features. The results are presented in **Tables 1** and **2**. Most RE-based models demonstrate improved accuracy compared to CONV models, with particularly notable gains in the SPA-CNN and SFS-CNN models. Specifically, OA, kappa, recall, and F1 scores improved by 8.57%, 0.0927, 0.0598, and 0.0609, and by 8.58%, 0.1019, 0.0923, and 0.093, respectively. The optimal RE model was SPA-BPNN, achieving OA, kappa, recall, and F1 scores of 92.86%, 0.9086, 0.9267, and 0.9263. These values represent improvements of 0.96%, 0.0118, 0.0102, and 0.0104, respectively, over the best-performing CONV model (SPA-BPNN).

**Table 1:** Performance evaluation results of *Erannis jacobsoni* disturbance detection models based on red edge features.

Dataset	Model	OA	Kappa	Recall	F1
RE	ANOVA-RF	86.19%	0.8280	0.8550	0.8551
	SPA-RF	88.10%	0.8502	0.8715	0.8714
	SFS-RF	90.95%	0.8849	0.9052	0.9050
	ANOVA-CNN	82.86%	0.7915	0.8358	0.8339
	SPA-CNN	84.76%	0.8098	0.8306	0.8290
	SFS-CNN	90.48%	0.8792	0.9016	0.9001
	ANOVA-BPNN	89.05%	0.8627	0.8911	0.8920
	SPA-BPNN	**92.86%**	**0.9086**	**0.9267**	**0.9263**
	SFS-BPNN	92.38%	0.9028	0.9230	0.9233

Bold values denote the best-performing model for each evaluation metric.

**Table 2:** Performance evaluation results of *Erannis jacobsoni* disturbance detection models based on conventional features.

Dataset	Model	OA	Kappa	Recall	F1
CONV	ANOVA-RF	88.57%	0.8563	0.8801	0.8798
	SPA-RF	87.14%	0.8394	0.8659	0.8638
	SFS-RF	89.05%	0.8620	0.8846	0.8844
	ANOVA-CNN	80.48%	0.7638	0.8124	0.8042
	SPA-CNN	76.19%	0.7171	0.7708	0.7681
	SFS-CNN	81.90%	0.7773	0.8093	0.8071
	ANOVA-BPNN	89.05%	0.8626	0.8934	0.8930
	SPA-BPNN	**91.90%**	**0.8968**	**0.9165**	**0.9159**
	SFS-BPNN	90.48%	0.8798	0.9058	0.9050

Bold values denote the best-performing model for each evaluation metric.

Among the three feature selection methods, selection based on SPA and SFS was the most effective. Taking the RE features as an example, the accuracy of SPA-RF improved by 1.91%, 0.0222, 0.0165, and 0.0163 compared with that of ANOVA-RF. The accuracy of SPA-BPNN improved by 3.81%, 0.0459, 0.0356, and 0.0343 in OA, kappa, recall, and F1, respectively, compared to ANOVA-BPNN. Although SPA-CNN showed slight decreases in recall (−0.0052) and F1 (−0.0049) due to omission errors compared to ANOVA-CNN, it achieved improvements of 1.9% and 0.0183 in OA and kappa, respectively. The accuracy of SFS-RF increased by 4.76%, 0.0569, 0.0502, and 0.0499 in OA, kappa, recall, and F1, respectively, compared to ANOVA-RF. The accuracy of SFS-CNN improved by 7.62%, 0.0877, 0.0658, and 0.0662 compared to ANOVA-CNN. The accuracy of SFS-BPNN improved by 3.33%, 0.0401, 0.0319, and 0.0313 compared to ANOVA-BPNN.

**Figure 7:**
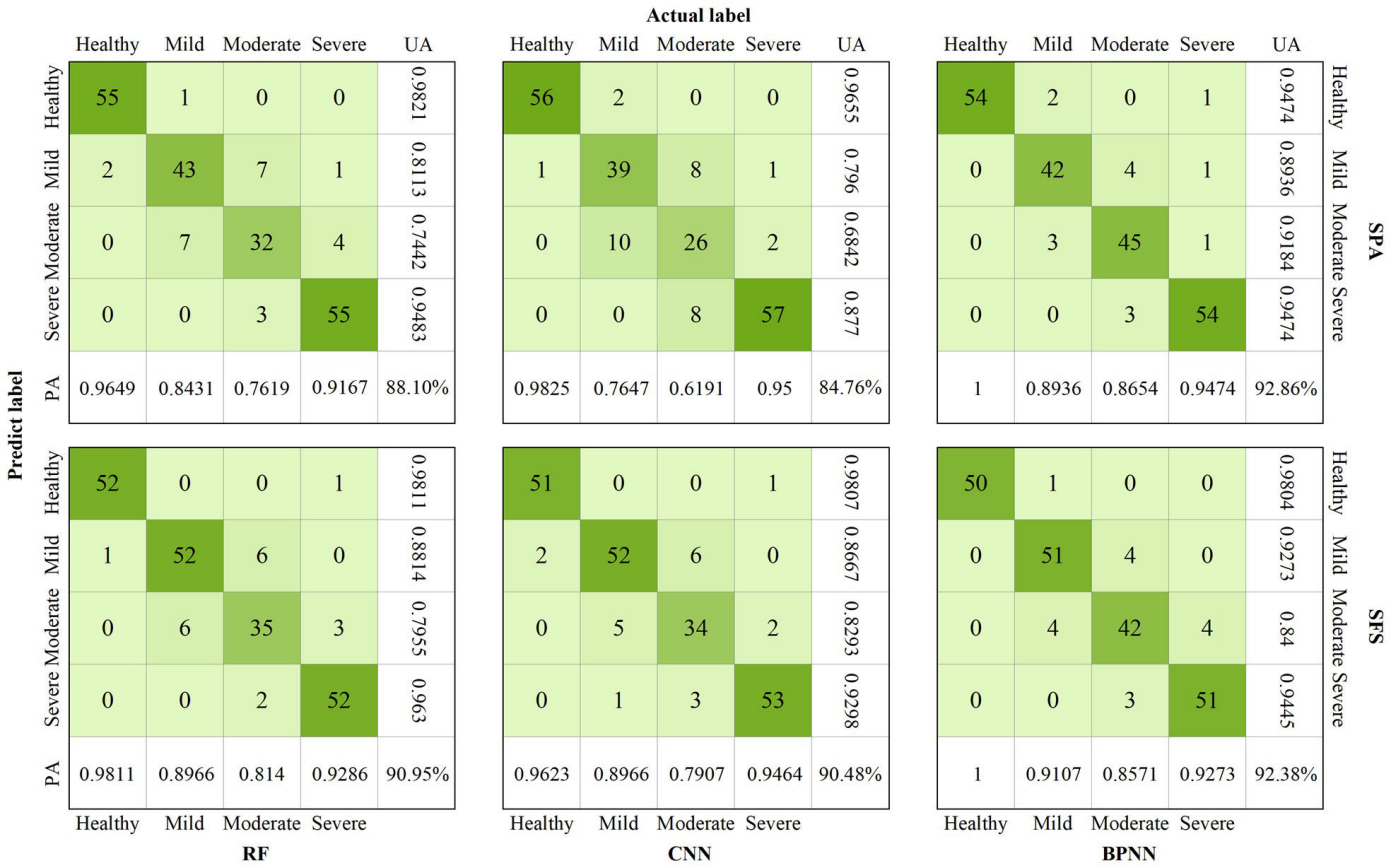
Confusion matrices of high-performance *Erannis jacobsoni* disturbance detection models: RF, CNN, and BPNN models developed using red edge features selected via SPA and SFS.

To examine the model detection performance at each damage level, the confusion matrices of the SPA and SFS models based on the RE features were plotted (**Figure 7**). The accuracy of each model reached more than 0.94 in the detection of healthy trees, especially in the BPNN model, where no misclassification occurred. A satisfactory accuracy of over 0.87 was achieved in detecting severe damage levels. Although there was slight confusion between mild and moderate levels, resulting in a decrease in accuracy, the performance still met the requirements for pest monitoring. Among the models, SFS-BPNN and SPA-BPNN models achieved optimal results in mild and moderate detections, respectively, with PA and UA values of 0.9107 and 0.9273 for mild, and 0.8654 and 0.9184 for severe.

### 3.3 Spatial distribution of pest disturbance

**Figure 8:**
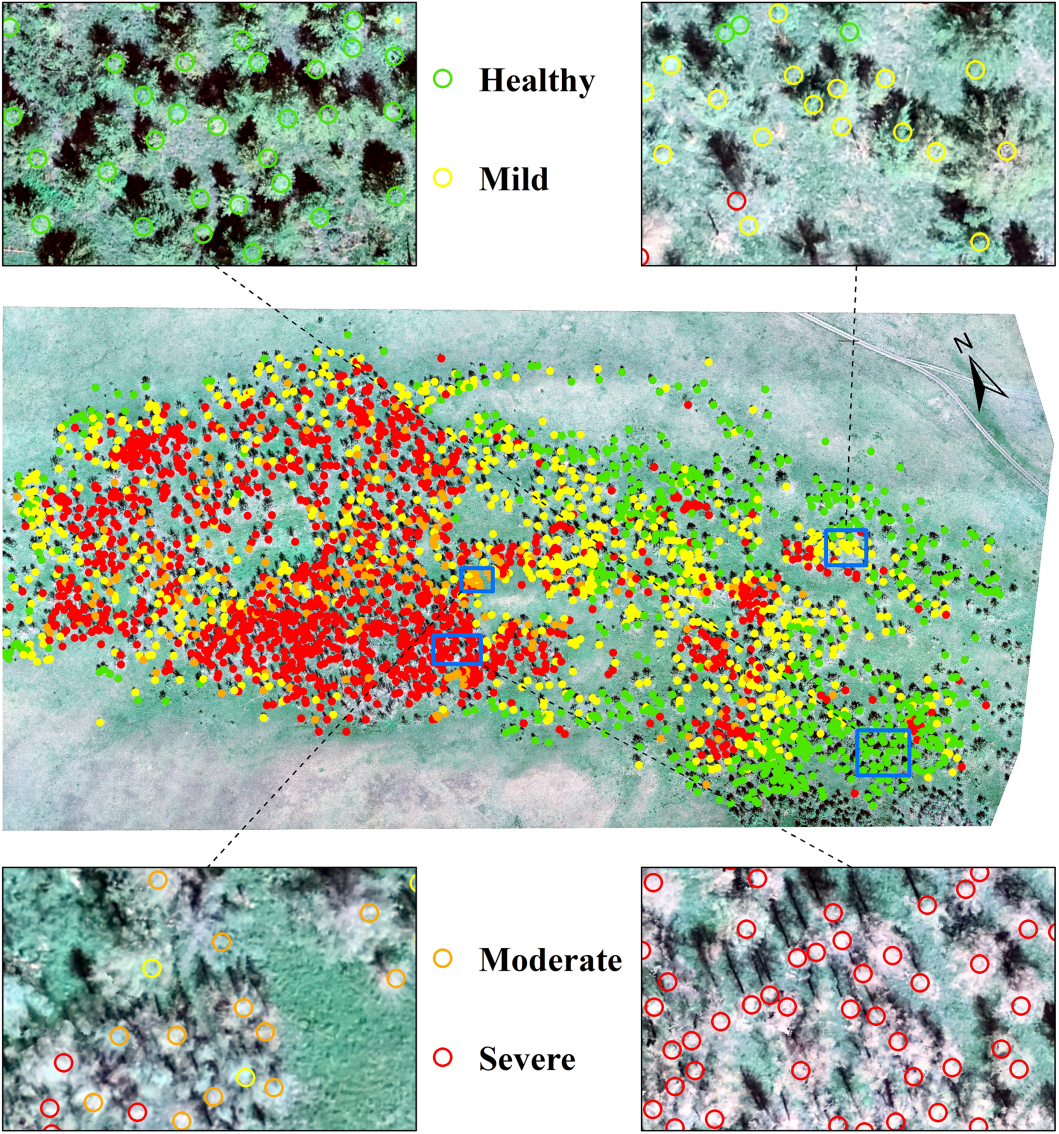
Spatial distribution of *Erannis jacobsoni* damage levels predicted by optimal model RE-SPA-BPNN, with magnified views of blue-framed regions.

To show the detection effect of this study more intuitively, based on the optimal model (RE-SPA-BPNN), the damage level of 2938 larch trees growing in the test area was inverted. The spatial distribution of the damage level of *E. jacobsoni* was then plotted (**Figure 8**). As a result, 685, 800, 221, and 1232 larch trees were found to be healthy, mildly, moderately, and severely damaged, accounting for 23.32%, 27.23%, 7.52%, and 41.93% of the total number of trees, respectively. Regarding spatial distribution, severe damage levels were concentrated in the southwestern part of the study area. Moderate levels were scattered among the severe levels, while mild and healthy levels surrounded the severe areas. Forest damage gradually decreased from the center of the study area outward, following a pattern of severe, moderate, mild, and healthy. This distribution was similar to the spatial pattern observed in predicting mulberry looper occurrences, as reported by Jia et al (Jia et al., 2019).

## 4 Discussion

### 4.1 Potential of RE features for pest identification detection

**Figure 9:**
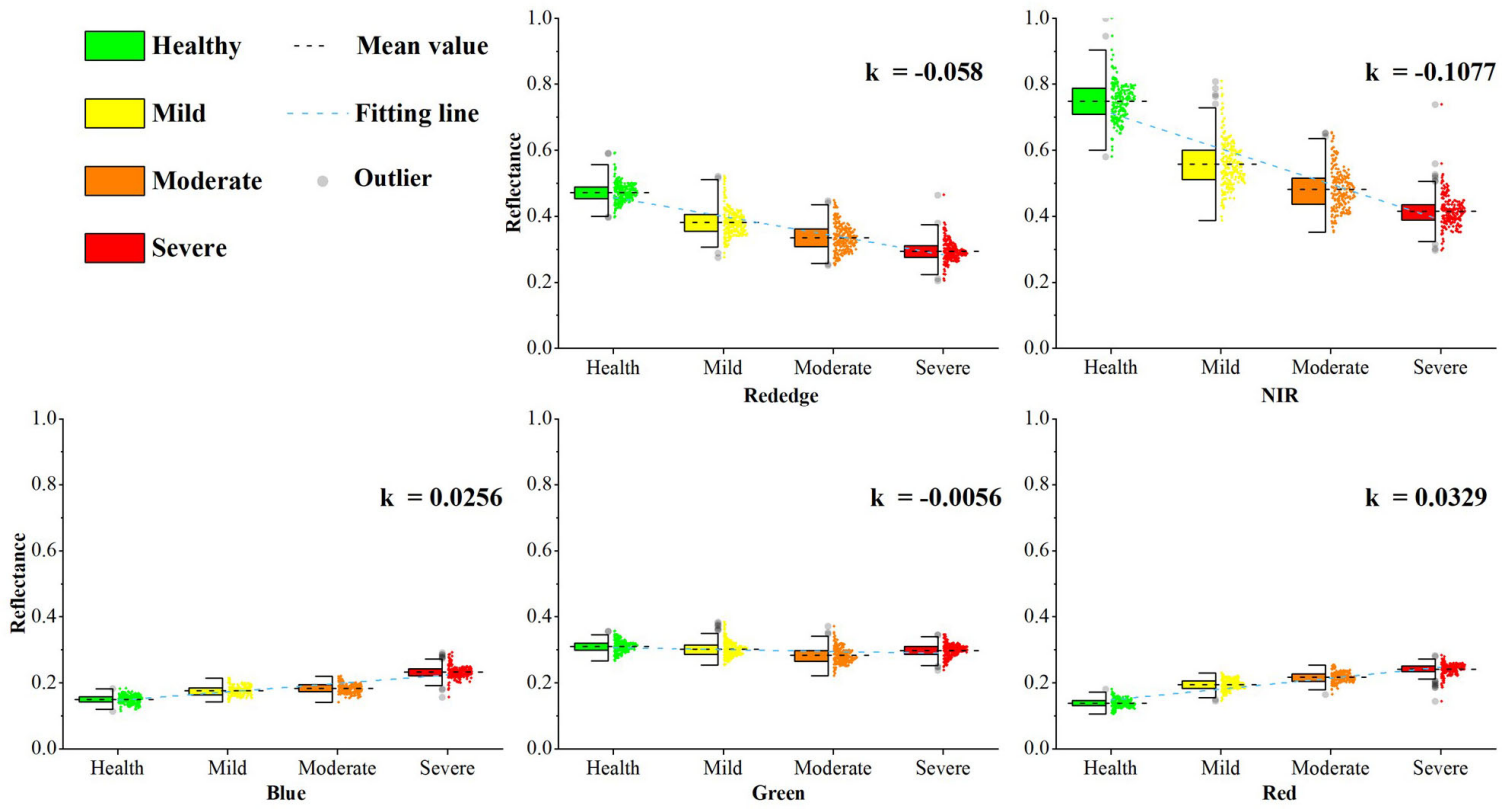
Canopy spectral (blue, green, red, red edge, NIR) response of Larix sibirica to *Erannis jacobsoni* damage levels, with trend slope *k* derived from linear regression of class-mean reflectance.

Previous studies have shown that outbreaks of *E. jacobsoni* infestations can lead to significant changes in the chlorophyll content of trees (Li et al., 2023). The RE band has been found to have advantages in measuring vegetation chlorophyll content (Lu et al., 2019; Bhattarai et al., 2020; Miao et al., 2025), and the results of this study on forest trees are consistent with these findings. To investigate the underlying reasons, the correlation graphs between the reflectance of each band and the level of damage in the canopy of samples were plotted (**Figure 9**). Linear fitting was performed based on the changes to analyze the degree of response of the spectral reflectance, where k is the slope, and R is the coefficient of determination. **Figure 9** shows that in the process of improving the damage level, the reflectance of the green band in the severe level increased instead of decreasing. This may be due to the high LLR of the trees at the severe level, which allowed the reflectance of the understory soil background to contribute to the canopy reflectance, thereby increasing the green band reflectance. The k values of the fitted curves for reflectance in the blue and red bands were 0.0256 and 0.0329, respectively, indicating a gradual increase with the decline in foliage volume and biochemical fractions of the trees. The k values of the fitted reflectance curves in the RE and NIR bands were 0.058 and 0.1077, respectively, showing a rapidly decreasing trend. This indicates that the RE and near-infrared (NIR) bands are more responsive to the changes in the pest damage levels. Among them, the RE reflectance fitting curves are flatter than those of the NIR band. However, the data at each damage level is more aggregated, with fewer outliers. The standard deviations of the data distributions at the four levels are lower than those of the NIR band by 0.02846, 0.02926, 0.02178, and 0.01702. This improves the detection effect, thus further affecting the derived RE features. Therefore, a better model was achieved by incorporating the RE band into the detection of *E. jacobsoni* damage levels. Similar results have been reported in the field of remote sensing detection, such as the conclusions reached by Schuster et al. (2012); Immitzer et al. (2016), and Zhang et al. (2017) in studies on crop classification, tree species identification, and land-use classification.

### 4.2 Impacts of feature selection methods on model accuracy

In this study, the feature data were downsized by three feature filtering methods. ANOVA has the smallest downsizing degree, and only two and one redundant features are eliminated from the original RE and CONV sets, respectively. This causes the ANOVA-based model to be less accurate. In addition, in ANOVA-RF, the poor performance of the RE features compared to the CONV features may be due to the model’s instability caused by overlapping data. While SPA eliminates 25 and 18 redundant features from the original RE and CONV sets, respectively, SFS removes seven CONV features and 25 RE features based on feature importance, yielding positive results. This is especially true for RE-based pest detection, where the OA consistently exceeded 84%. The complexity of vegetation indices’ responses to pest damage levels cannot be described by a simple linear relationship. ANOVA focuses on linear problems and tends to exclude feature correlations, making it difficult to optimally extract features (Xu et al., 2022b). Meanwhile, SPA and SFS can analyze both linear and nonlinear complex patterns; the correlation between features can be considered by feature covariance minimization and importance, respectively (Rogers and Gunn, 2006; Zeng and Ye, 2012; Sauwen et al., 2017; Schonlau and Zou, 2020). Features are added gradually from a small number of features in a forward selection manner, and the optimal subset of features is then obtained to improve model accuracy.

**Figure 10:**
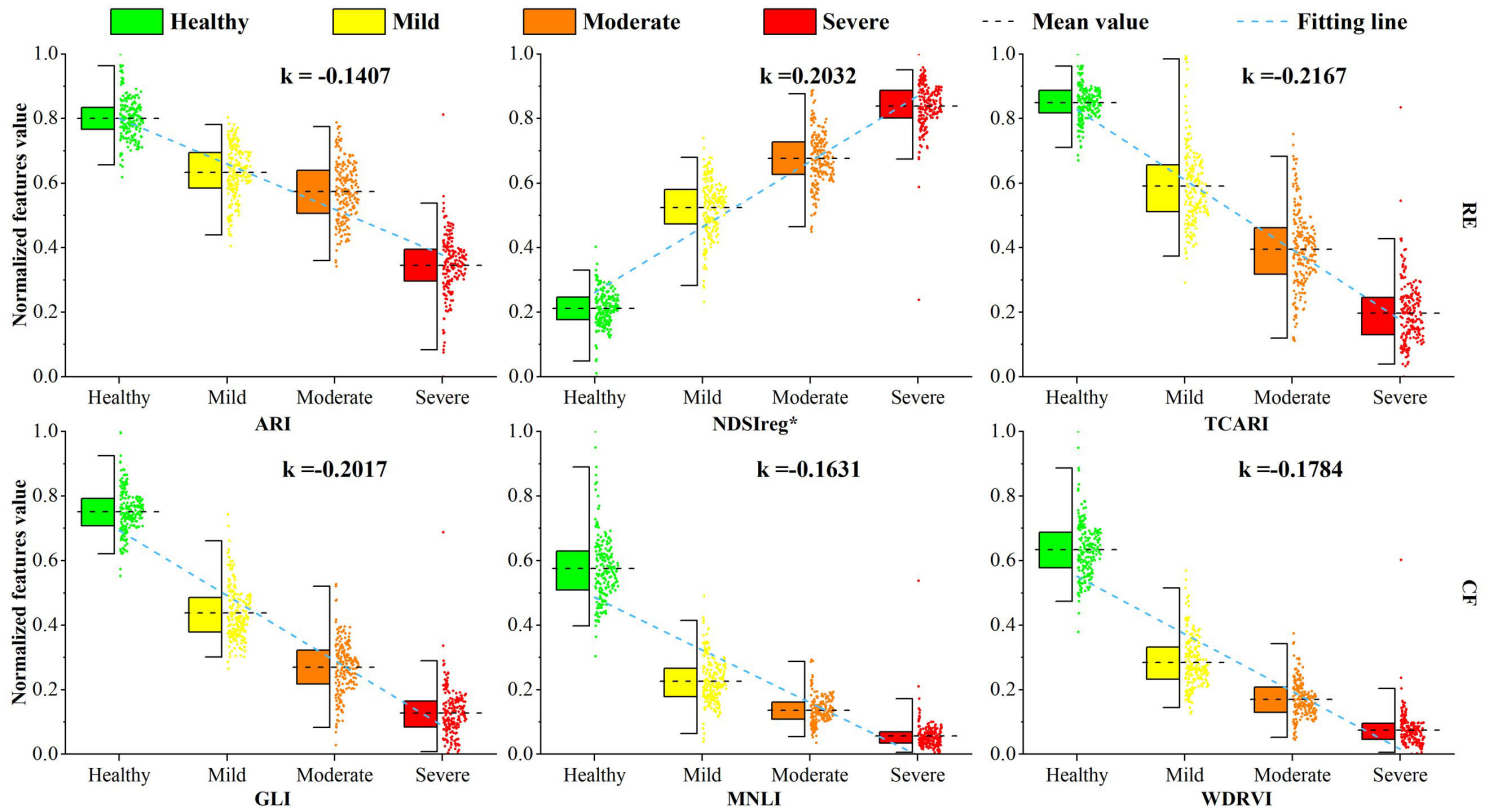
Canopy response of conventional and red edge features in *Larix sibirica* to *Erannis jacobsoni* damage levels, with slope *k* derived from linear regression of class-mean feature values.

In the process of selecting the sensitive RE subset, ARI, NDSIreg*, and TCARI were selected by both SPA and SFS. All had F-values over 1000 in the ANOVA (**Figure 5**). Among them, ARI is extremely sensitive to the anthocyanin and pigment content of trees and can characterize the pigment uptake state of affected trees (Gitelson et al., 2006; Skoneczny et al., 2020). It was also selected as an effective index for detecting tree defoliation in the study by Bhattarai et al. (2020). NDSIreg* can characterize the chlorophyll and water uptake status of needles in damaged trees based on the composition of red and red edge band differences, showing strong correlation with chlorophyll content in Bai et al. (2023) research. TCARI is extremely sensitive to changes in chlorophyll during vegetation damage (Zarco-Tejada et al., 2005), and demonstrated particular utility in Wu et al. (2021) study. Taking these three indices as examples, we plotted the variation of red-edge (RE) feature values with damage severity and compared them against the three optimal CONV features (GLI, MNLI, WDRVI) (**Figure 10**). The slopes of change increased after the features were constructed using the RE band, particularly for NDSIreg* and TCARI, which exceeded 0.2. This represented a significant improvement compared to the CONV features. The slope of change in ARI was lower, likely because it is an auxiliary feature that provides supplementary information related to anthocyanins for other RE features. Its contribution to pest detection is relatively small but essential. These results are consistent with the results of Chang et al. (2020), who demonstrated that RE indices can more effectively reveal spectral differences between healthy and infested plants compared to CONV indices. However, our results diverge from Guerra-Hernández et al. (2021) UAV-based tree health classification study, where CONV features outperformed RE features. This discrepancy may stem from the narrow bandwidth (10 nm) of the red edge band in their sensor, potentially limiting detection of pigment-driven spectral variations. Similarly, Santos et al. (2022) observed superior discriminatory performance in green and near-infrared (NIR) bands over red-edge features for plant-nematode damage detection. This outcome may be attributed to two factors: the narrow red-edge bandwidth (10 nm) and the established principle that red-edge bands exhibit greater effectiveness in high-biomass vegetation (Curran et al., 1990). The lower biomass of their target plants relative to forest ecosystems likely contributed to these divergent results.

### 4.3 Model performance evaluation

**Figure 11:**
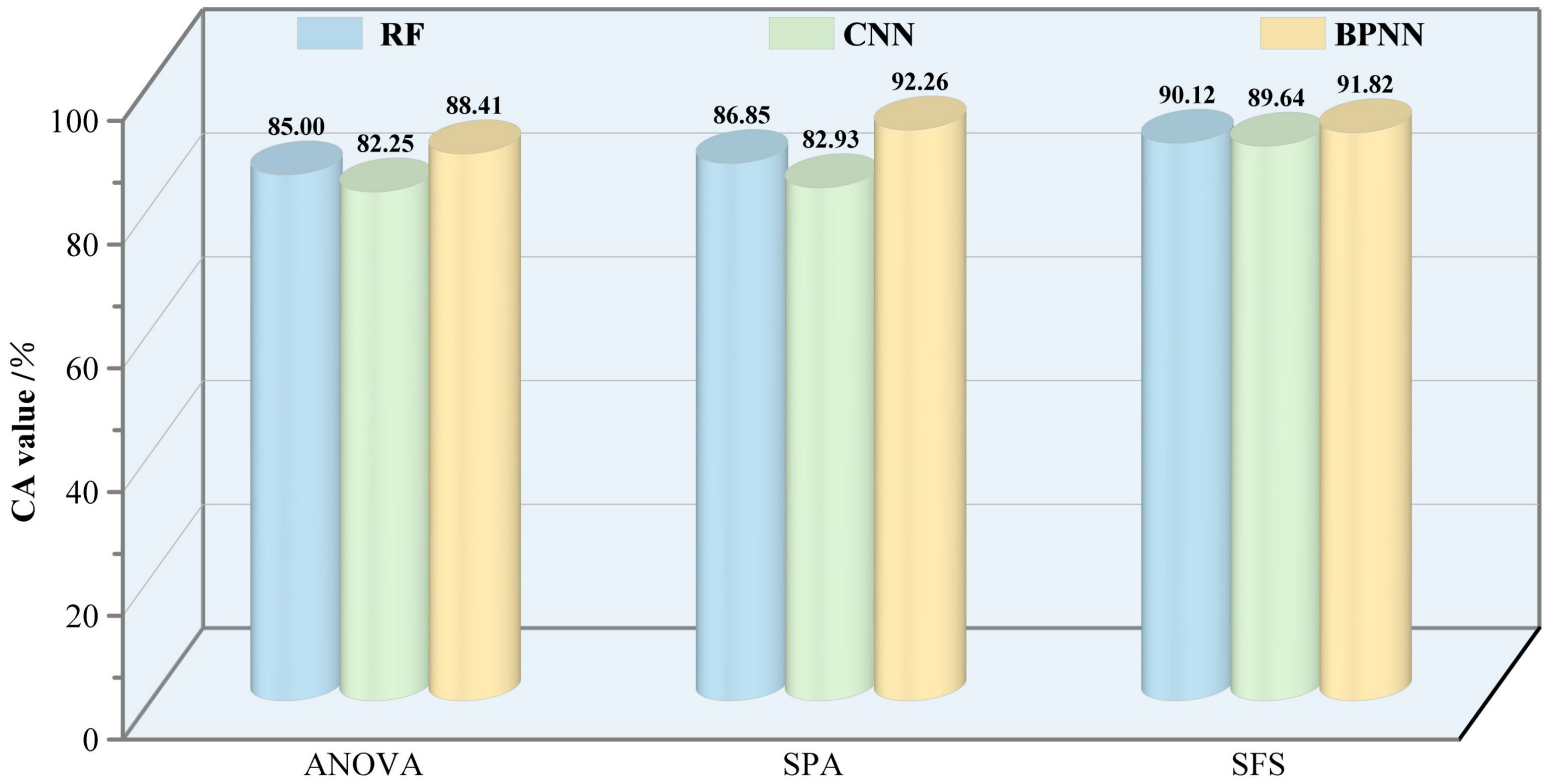
Comprehensive accuracy assessment of *Erannis jacobsoni* infestation detection using red edge-based modeling.

In this study, based on RE features, three widely applied algorithmic models were used to detect the damage level of *E. jacobsoni*. These algorithms have been proven effective in prior studies on pest infestations (Ma et al., 2019; He et al., 2025). Specifically, RF offers high-precision classification through ensemble learning, CNN excel at extracting intricate local spatial features, and BPNN are advantageous in identifying nonlinear relationships. By applying models with diverse operational principles, the likelihood of successfully detecting vegetation damage or pest infestations is increased (Miao et al., 2025., He et al., 2025). To evaluate the potential of the three models more intuitively, the four evaluation indicators were combined into one indicator, CA, with the same weights. The results are shown in **Figure 11**. BPNN is the most effective across different sensitive datasets, with CA of up to 92.26% (SPA-BPNN), followed by RF with a CA of 90.12% (SFS-RF). CNN is slightly less effective, with the highest CA of 89.64% (SFS-CNN), but it is still fully capable of being used in pest detection studies. In general, deep learning outperforms traditional machine learning in classification and recognition tasks (Liu and Wang, 2021). However, the performance of CNN in this study is slightly lower compared to RF, and CNN reduces by 2.75, 3.92, and 0.48% compared to RF in the sensitive datasets based on ANOVA, SPA, and SFS, respectively. This is partly due to the fact that while CNN can implicitly learn features from training data, it still faces the challenge of feature selection when working with a relatively small sample size. A sufficient sample size is often difficult to obtain, which may affect its classification performance (Yu et al., 2021; Alzubaidi et al., 2021). In this study, a detection model was constructed through a one-dimensional vector relationship between RE features and the level of damage, while a CNN is more suitable for damage-level recognition directly from visible-light images in a multidimensional perspective (Sudowe and Leibe, 2011).

### 4.4 Patterns of spatial distribution of pests

**Figure 12:**
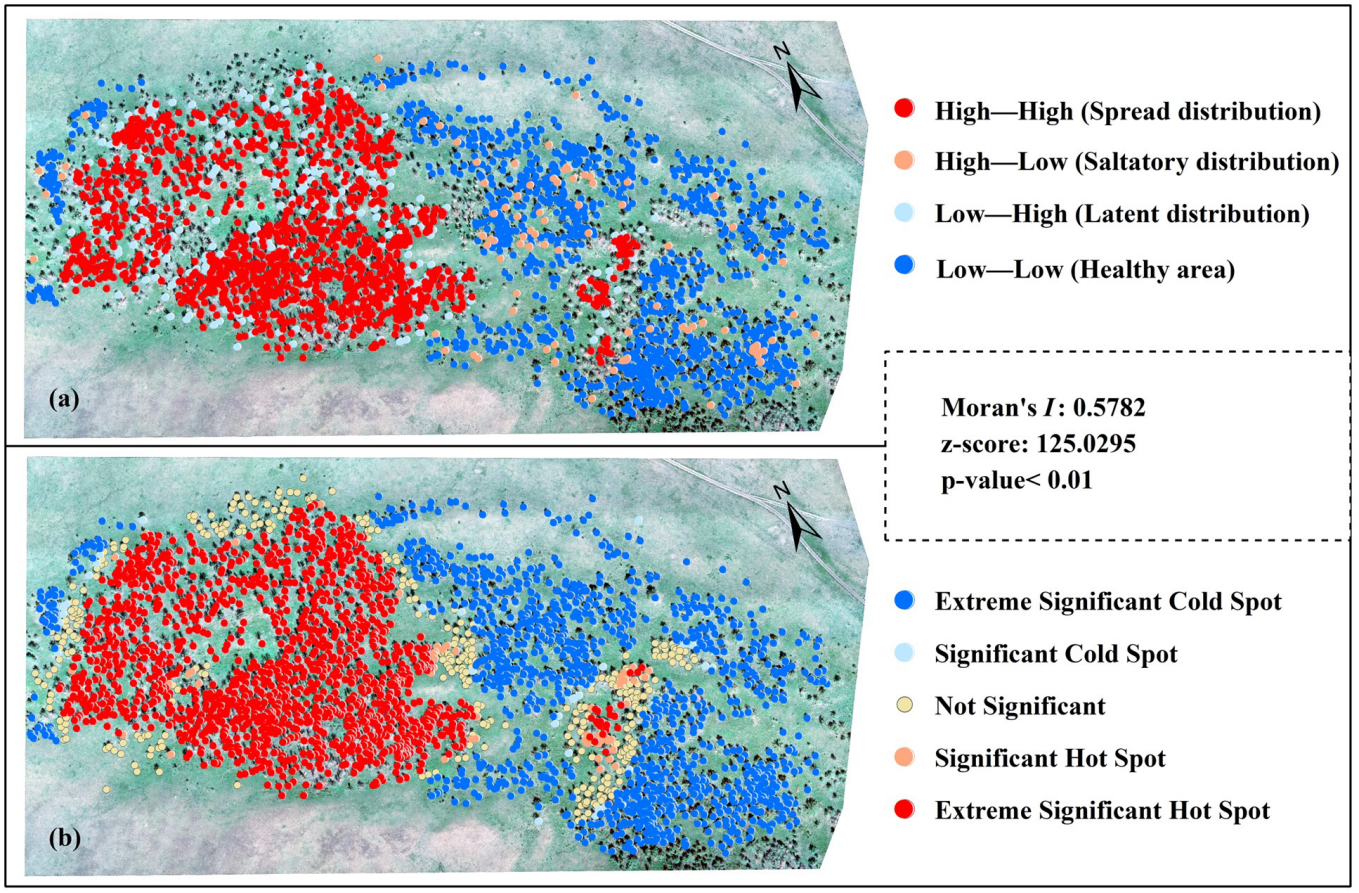
Spatial autocorrelation visualization of predicted *Erannis jacobsoni* damage distribution from optimal model. **(a)** Anselin Local Moran’s I (for cluster-outlier detection); **(b)** Getis-Ord Gi*(for hot spot identification).

Herein, we realized the inversion of the spatial distribution of the damage level of *E. jacobsoni* using an optimal model. Based on the Global Moran’s I value, Anselin Local Moran’s I, and Getis-Ord Gi*, indicators of global and local statistics (Moran, 1948; Anselin, 1995; Getis and Ord, 2010) further revealed the spatial correlation and clustering of *E. jacobsoni* disturbances (**Figure 12**). In terms of global statistics, a significant positive autocorrelation in the overall spatial distribution of pest damage levels can be inferred from Moran’s I, Z-value, and P-value, as shown in **Figure 12**. For local statistics, the two indicators consistently characterized the aggregation of pest damage levels at the local scale. Using Anselin’s Local Moran’s I, this study classified the four infestation types—high–high, high–low, low–high, and low–low—into spread distribution, saltatory distribution, latent distribution, and healthy areas, respectively (**Figure 12a**). Most of the infested areas were dominated by a spread distribution. This is consistent with what was mentioned by Weseloh (Weseloh, 1989) and was distributed in the northwestern part of the experimental area. Meanwhile, the healthy areas surrounded the spread distribution, which was mainly distributed in the southeastern part of the experimental area. This indicated that the diffusion pattern of the infestation spread outward from the aggregated centers gradually to the healthy areas.

Saltatory and latent distributions were scattered in the spread distribution and healthy areas, respectively. This may be attributed to factors such as wind force and wind direction, which affect diffusion patterns and result in the saltatory distribution of the pest. The strong resistance of some forest trees temporarily reduces the infestation rate, resulting in delayed infestation and allowing damage to be distributed latently (He and Alfaro, 1997, 2000). For Getis-Ord Gi* (**Figure 12b**), the extremely significant hotspots—areas with high damage values —were mainly clustered in the central to northwestern part of the study area. These hotspots were surrounded by sporadic significant hotspots and, further outward, by areas of low or no damage, which can be considered spatially clustered cold spots. This pattern is consistent with the results of the inversion. The reason for this is that, according to the field investigation, *E. jacobsoni* uses the soil under the canopy of the larch as the egg-laying site during reproduction. After the larvae from the originating site have passed the incubation period and drilled out of the soil, they take the larch as the host and feed on its needles. E. jacobsoni is not a particularly mobile insect. When its host trees are severely damaged and lack needles, the insect spits silk and either falls to the ground or is carried by the wind. It can also crawl to nearby trees, where it begins to feed on the new needles until they are completely consumed. This behavior causes a regular spatial change in the level of damage. Therefore, it is recommended that forestry managers utilize the current spread patterns of *E. jacobsoni*, in conjunction with field surveys and monitoring data, to implement targeted control measures such as pesticide spraying, fumigation, and biological control. Additionally, employing aerial application methods for radial control along the pest’s dispersal direction and establishing pheromone-based trapping networks can enhance management efficacy. This integrated strategy aims to reduce labor and financial inputs, lower operational costs, and achieve cost-effective pest management.

### 4.6 Challenges and prospects

This study was based on single-time-phase UAV multispectral imagery to identify disturbances in the pre-mid phase of pest infestation and obtain ideal results. Nevertheless, detection accuracy for early-stage infestations (mild level) remains suboptimal, primarily due to minimal spectral separability between healthy and mildly stressed vegetation—a critical challenge requiring resolution. Notably, integrated approaches incorporating thermal infrared, fluorescence, and radar data with optical remote sensing have gained traction among researchers, proving instrumental in generating more reliable results (Lin et al., 2019; Duarte et al., 2022; Zhang J. et al., 2019). Concurrently, multi-angular remote sensing techniques represent a promising strategy worthy of exploration to improve pest detection efficiency (Li et al., 2015). Therefore, multisource data fusion and multi-angle aerial photography schemes are expected to be used in future experiments. In terms of the spatial distribution of pests, under high population density conditions, competition and movement should reduce aggregation and approach randomness (Waters, 1959; Taylor, 1984). If aggregation behavior does not decrease with increasing density, populations are behaviorally limited (Henson, 1959). During the outbreak phase, the spatial distribution pattern of the infestation changes as it shifts from low to high-density populations. However, single-time-phase data acquisition constrains observation of spatiotemporal infestation patterns throughout developmental cycles. Consequently, we will incorporate multitemporal images during the infestation period into our future experiments to analyze the spatial distribution of *E. jacobsoni* in more detail. Moreover, while drone-based remote sensing has been employed for pest detection, it exhibits significant limitations in applications requiring time series analysis and large-scale monitoring. These limitations include a scarcity of historical data, the substantial financial and labor costs associated with acquiring long-term continuous datasets, and the inherent challenges in obtaining large-scale data. Consequently, satellite remote sensing is often more suitable for studies necessitating extensive spatial and temporal coverage (Luo et al., 2023). Nevertheless, data derived from UAV can provide essential training and validation samples. When integrated with satellite imagery, this approach enables enhanced precision in pest detection across broader spatial scales, offering valuable insights for large-scale pest management strategies.

## 5 Conclusion

In this study, we utilized UAV images and field survey data of the origin of the *E. jacobsoni* infestation to derive the RE spectral features, and integrated feature extraction and machine learning algorithms to detect disturbances caused by the *E. jacobsoni*. Results demonstrate that SPA and SFS exhibited exceptional feature selection capabilities: SPA identified 6 RE and 13 CONV features, while SFS selected 6 RE and 24 CONV features, with these optimized combinations proving effective for *E. jacobsoni* detection. Most RE-based models showed superior accuracy to CONV features, reaching peak overall accuracy of 92% (vs. 91% for optimal CONV performance) while demonstrating robust pest detection capabilities. After inverting the damage levels of all trees using this model, the spatial autocorrelation was analyzed. It was found that *E. jacobsoni* exhibited a clumped distribution, with damage levels gradually decreasing from the point of origin outward. This pattern is linked to the life habits of the pests.

Overall, this study has shown the advantages of RE features in pest detection, aiming to provide an experimental basis for the development and application of detection technology in pest control and contribute to a solid foundation for the next step of large-scale and high-precision pest monitoring, early warning, and familiarization with the occurrence pattern.

## Data Availability

The original contributions presented in the study are included in the article/**Supplementary Material**. Further inquiries can be directed to the corresponding author.

## Supplementary material

Supplementary Figure 1
**(a)** Spatial distribution of samples: green dots represent healthy trees, yellow dots indicate mild damage, orange dots show moderate damage, and red dots denote severe damage. **(b)** Field photographs of sample trees (*Larix sibirica*) affected by *Erannis jacobsoni* at different damage levels. **(c)** The UAV utilized in this study for capturing images of the *Erannis jacobsoni* occurrence area: DJI Phantom 4 Multispectral Version, along with its sensor parameters.

Supplementary Figure 2 Evaluation results of the *Erannis jacobsoni* disturbance detection model using conventional features based on comprehensive accuracy.

Supplementary Table 1 Spectral indices used in the development of the *Erannis jacobsoni* detection model.
