# Supplementary figures and images for: Erannis jacobsoni disturbance detection based on unmanned aerial vehicle red edge spectral features
Source: Front Plant Sci. 2025 Sep 1;16:1619695. doi: 10.3389/fpls.2025.1619695 (PMC12434972; doi:10.3389/fpls.2025.1619695)

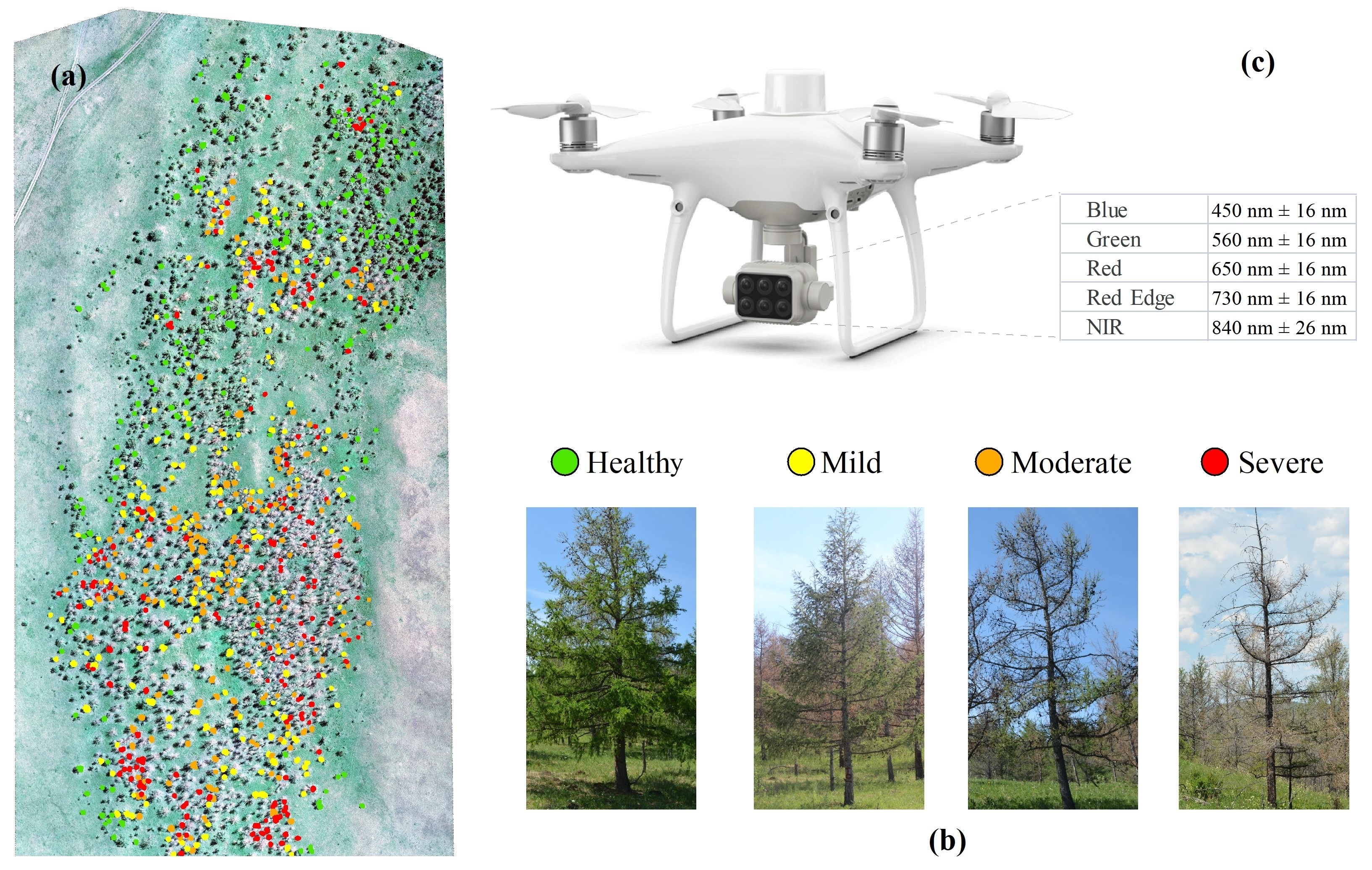

Supplement: Supplementary Figure 1 — (a) Spatial distribution of samples: green dots represent healthy trees, yellow dots indicate mild damage, orange dots show moderate damage, and red dots denote severe damage. (b) Field photographs of sample trees (Larix sibirica) affected by Erannis jacobsoni at different damage levels. (c) The UAV utilized in this study for capturing images of the Erannis jacobsoni occurrence area: DJI Phantom 4 Multispectral Version, along with its sensor parameters. [file Image1.jpg]

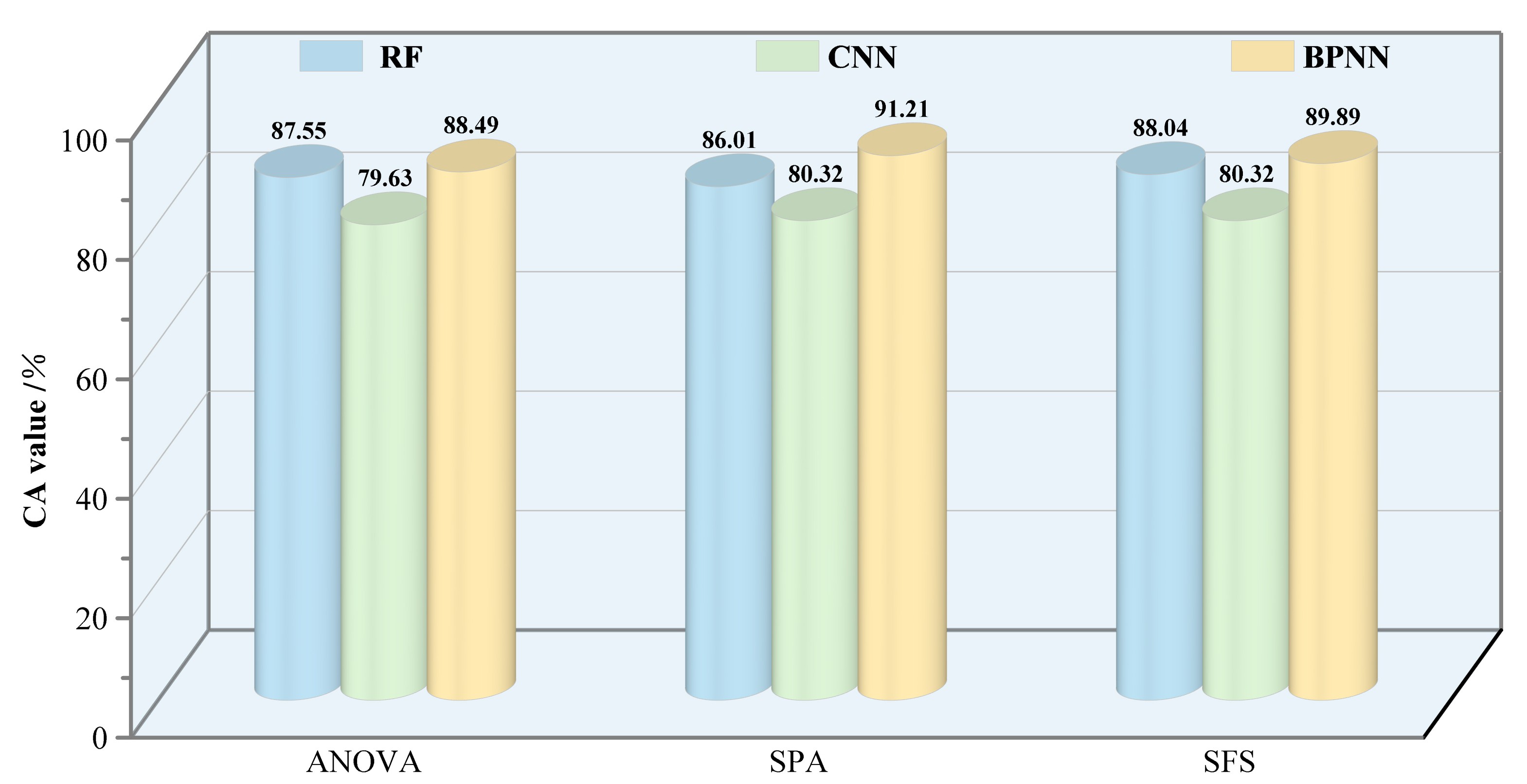

Supplement: Supplementary Figure 2 — Evaluation results of the Erannis jacobsoni disturbance detection model using conventional features based on comprehensive accuracy. [file Image2.jpg]
