# Supplementary material for: Erannis jacobsoni disturbance detection based on unmanned aerial vehicle red edge spectral features
Source: Front Plant Sci. 2025 Sep 1;16:1619695. doi: 10.3389/fpls.2025.1619695 (PMC12434972; doi:10.3389/fpls.2025.1619695)
Supplement: Supplementary Table 1 — Spectral indices used in the development of the Erannis jacobsoni detection model. [file Table1.docx]

**Supplementary table S1.** Spectral indices used in the development of the *Erannis jacobsoni* detection model.

| Conventional features | Formula | Red edge features | Formula |
| --- | --- | --- | --- |
| DVI | NIR-Red | CIreg | （NIR-RE）/(NIR-Red) |
| GDVI | NIR-Green | NDRE | (NIR-RE)/(NIR+RE) |
| TVI | 0.5[120(NIR-Green)-200(Red-Green)] | DVIreg | NIR-RE |
| RVI | NIR/Red | RVIreg | NIR/RE |
| GRVI | NIR/Green | EVIreg | 2.5(NIR-RE)/(NIR+6RE-7.5Blue+1) |
| CIGreen | NIR/Green-1 | Int1reg | (Green+RE)/2 |
| RCI | NIR/Red-1 | Int2reg | (Green+RE+NIR)/2 |
| NDVI | (NIR-Red)/(NIR+Red) | SIreg | (Blue+RE)^0.5^ |
| GNDVI | (NIR-Green)/(NIR+Green) | SI1reg | (Green*RE)^0.5^ |
| RDVI | (NIR-Red)/(NIR+Red)^0.5^ | SI2reg | (Green^2^+RE^2^+NIR^2^)^0.5^ |
| GRDVI | (NIR-Green)/(NIR+Green)^0.5^ | SI3reg | (Green^2^+RE^2^)^0.5^ |
| NLI | (NIR^2^-Red)/(NIR^2^+Red) | NDSIreg | (RE-NIR)/(RE+NIR) |
| 2NLI | (NIR^2^-Green)/(NIR^2^+Green) | OSAVIreg | (NIR-RE)/(NIR+RE+0.16) |
| MSR | (NIR/Red-1)/[(NIR/Red)^0.5^+1] | RDVIreg | (NIR-RE)/(NIR+RE)^0.5^ |
| GMSR | (NIR/Green-1)/[(NIR/Green)^0.5^+1] | NDVIreg | (NIR-RE-0.045×Green) /( NIR-RE + 0.045×Green) |
| CI | [NIR/Green-1]/(Green/Red) | ARI | (1/Green)-(1/RE) |
| GMNLI | 1.5(NIR^0.5^-Green)/(NIR^0.5^+Green+0.5) | DVIreg* | RE-Red |
| SAVI | 1.5(NIR-Red)/(NIR+Red+0.5) | Intreg* | (RE+Red)/2 |
| GSAVI | 1.5(NIR-Green)/(NIR+Green+0.5) | Int2reg* | (Green+Red+RE)/2 |
| OSAVI | (NIR-Red)/(NIR+Red+0.16) | RECI | NIR/RE-1 |
| GOSAVI | (NIR-Green)/(NIR+Green+0.16) | SIreg* | (RE+Red)^0.5^ |
| MTVI2 | 1.5[1.2(NIR-Green)-2.5(Red-Green)]/[(2NIR+1)^2^-(6NIR-5Red^0.5^)-0.5]^0.5^ | SI1reg* | (RE*Red)^0.5^ |
| MSAVI2 | {2NIR+1-[(2NIR+1)^2^-8(NIR-Red)]^0.5^}/2 | SI2reg* | (Green^2^+Red^2^+RE^2^)^0.5^ |
| SCCI | lnRed/[(NIR-Red)/(NIR+Red)] | SI3reg* | (RE^2^+Red^2^)^0.5^ |
| NDGI | (Green-Red)/(Green+Red) | EVIreg* | 2.5(RE-Red)/(RE+6Red-7.5Blue+1) |
| WDRVI | (0.1NIR-Red)/(0.1NIR+Red) | RVIreg* | RE/Red |
| MNLI | 1.5(NIR^0.5^-Red)/(NIR^0.5^+Red+0.5) | MSRreg | (NIR/RE-1)/(NIR/RE+1)^0.5^ |
| lnRE | 100（lnNIR-lnRed） | TCARI | 3[(RE-Red)-0.2(RE-Green)(RE/Red)] |
| GMSR | (NIR/Green-1)/[(NIR/Green)^0.5^+1] | NDVIreg* | (RE-Red)/(RE+Red) |
| EVI | 2.5(NIR-Red)/(NIR+6Red-7.5Bllue+1) | LCI | (NIR-RE)/(NIR+Red) |
| GLI | [(Green-Red)+(Green-Blue)]/2Green+Red+Blue | NDSIreg* | (Red-RE)/(Red+RE) |

*: Blue: Blue band, Green: green band, Red: Red band, RE: Red edge band, NIR: Near-infrared band.
